# A Systems Dynamic Approach to Alzheimer's Disease Prevention

**DOI:** 10.23889/ijpds.v1i1.397

**Published:** 2017-04-19

**Authors:** Nasim S. Sabounchi

**Affiliations:** 1 State University of New York at Binghamton

## Objectives

As estimated there are about 5.3 million who suffer from Alzheimer's disease in United States. The incidence is increasing as the population is aging. Due to the increasing trend of Alzheimer's disease, there is a lot of discussion on prevention efforts or slowing the incidence. Also, models that could predict individual risk of cognitive impairment are needed to assist in prevention efforts.

In general dementia development has been associated with growth in various vascular, lifestyle and other risk factors. Epidemiological research provides evidence of some vascular, lifestyle and psychological risk factors that are modifiable and protective of disease incidence either independently or while interacting with other factors. However, as reported by National Institute of Aging, it is not yet clear whether health or lifestyle factors can prevent Alzheimer's disease.

The objective of this research project is to adopt a system dynamics modeling approach to study the interaction of several key factors including vascular, lifestyle and psychological aspects over the life course of individuals, to gain further understanding of Alzheimer's disease incidence and evaluate prevention strategies. Both datasets of `Alzheimer's Disease Neuroimaging Initiative (ADNI)' and `Health and Retirement Study (HRS)' will be used for model development and validation.

## Approach

A system dynamics approach is an optimal choice for addressing the goal of this proposal because different key factors interact over time and make Alzheimer's disease incidence a complex problem. Furthermore, system dynamics approaches focus on understanding the relationship between the structure of a system and the resulting dynamic behaviors generated through multiple interacting feedback loops. Such an approach could be invaluable in studying dynamic problems arising in complex health, social, economic, or ecological systems.

## Results

For the purpose of the proposal, the following stages are planned:

1. Develop a system dynamics simulation model at individual level that predicts the Alzheimer's disease incidence over the life course, and aggregates individual level models to predict population level trends
2. Calibrate the resulting simulation model based upon longitudinal data trends employed from Alzheimer's Disease Neuroimaging Initiative (ADNI) database. Both cohorts with Alzheimer's disease and control subjects from this database will be used to fine-tune the simulation model.

## Conclusion

The final validated model would be used to test different hypotheses and evaluate various strategies and/or their combinations to help evaluate the efficacy of prevention strategies on Alzheimer's disease incidence and its growth.

